# Cummings' Patent vs. Dr. T. B. Gunning

**Published:** 1872-12

**Authors:** 


### EDITORIAL DEPARTMENT.

---

*Cummings' Patent vs. Dr. T. B. Gunning.*—For the information of the dental profession, we send you for publication, a copy of the answer used by Dr. T. B. Gunning, Dentist, of New York city, as defendant in suit brought against him by the Goodyear Dental Vulcanite Company, for infringement of the Cummings' patent, minus Schedule A, which is a reply to the allegations as to the Gardner decision, recited in the Goodyear Dental Vulcanite Company's bill against him, and for prudential reasons will not be published out of a suit.

This is the fourth suit that has been brought against Dr. Gunning in the past six years, and this last one was dismissed at cost of plaintiff. They had failed in six years litigation of the matter to find out that the Dr. was entitled to the privilege of their patents, but suddenly the Dr's. answer, which they would not allow to go on record, for fear it would compromise their interests in instigating others to contest, convinced them that Dr. Gunning was, and had been all the time, entitled to these privileges. There is in our opinion, nothing to prevent the dentists from defending themselves against the Cummings' patent, except want of unity.

Dr. S. S. White, of Philadelphia, upon the affidavits on file, as published in the November *Cosmos*, has made application through his counsel, Messrs Black & Baldwin, of Pennsylvania, to the Supreme Court of the United States, for a reversal of their decision, in the Gardner Appeal Case, rendered the 6th day of last May. The motion will be argued at an early day, and we have every reason to believe that it will have a favorable consideration.

R. B. Winder, D. D. S.,
*Chairman Executive Committee.*

State of New York,
City and County of New York, } *ss.*

Thomas B. Gunning, being duly sworn, deposes and says: That he is a Dentist and Dental Surgeon, doing business in the city of New York; that on or about the 7th day of June, 1872

a suit in equity was begun against him in the Circuit Court of the United States for the Southern District of New York, by the Goodyear Dental Vulcanite Company of New York, and one Josiah Bacon, for alleged infringement of the letters patent granted to John A. Cummings, June 7th, 1864, and re-issued to the Dental Vulcanite Company, of Boston, (the assignor of the complainants,) January 10th and March 21st, 1865:

That the bill of complaint in said suit, among other things, contained the following allegation:

* * * * Your orators further show unto your honors, that one Benoni E. Gardner, a citizen of the State of Rhode Island, infringed upon the said patent, (meaning the patent herein above described by this deponent,) whereupon your orators filed their bill of complaint against said Gardner, on the 16th day of September, 1868, in the United States Circuit Court, for the District of Rhode Island, praying for an injunction and other relief in equity against said Gardner; to which said bill of complaint said Gardner filed his answer, alleging, among other defences, that the said Cummings was not the original and first inventor of said improvement, and setting up that many other persons had used and practised the said improvement before the invention thereof by said Cummings, as claimed and set forth in said bill of complaint. And thereupon, evidence was fully and exhaustively taken by both parties upon all the points at issue, and the cause was brought to a final hearing on its merits and fully argued before said Court, before Hon. Nathan Clifford, Judge, and thereupon, at the June term, 1870, the judgment of said Court was pronounced in favor of the complainants on all the points at issue, and a decree was entered directing, among other things, that a perpetual injunction be issued against said Gardner, enjoining him from infringing upon said letters patent. And thereupon, the said Benoni E. Gardner appealed from said decree to the Supreme Court of the United States, and the said Supreme Court, after full argument by counsel for both parties, rendered its decision affirming said decree, on the 6th day of May, 1872.

That on the 5th day of October, 1872, the complainants' solicitors, by stipulation, extended deponent's time for filing his answer to the said bill of complaint to Oct. 14th, and that in consideration of said extension, this deponent agrees to deliver in advance of the filing thereof, and on the 9th of October did so deliver to the complainants, said solicitors, for perusal, a copy of his intended answer, then nearly completed, and which was thereafter completed without any substantial alteration.

That the main defences stated in said answer were as follows:

I. That John A. Cummings was not the inventor of the improvement claimed by his patent, or of any material part of it.

II. That admitting him to have made such invention he had abandoned and dedicated the same to public use.

III. That the re-issued patents of January 10th and March 21st, 1865, above mentioned, were void for unlawfully enlarging the claim of the original patent.

IV. That the process given, or intended to be given, by all of said letters patent, was improperly specified, and that by said process, the improvement claimed by the patent could not be manufactured red so as to be applicable to dental purposes.

V. Besides these defences upon the merits, the further partial defence that this deponent, by virtue of a certain license under the re-issued patents of Nelson Goodyear, granted to his administrator, Henry B. Goodyear, May 18th, 1858, and by certain arrangments between this deponent's licensors and the assignors of the complainants, was entitled to continue unmolested by the holders of the Cummings' patent, up to the 6th day of May, 1872.

That in response to the allegations herein above granted, from the bill of complaint against him, his deponent in his said answer, made the averments set forth *verbatim* in Schedule A hereunto annexed. And in relation to said averments, deponent further specifically says that he then believed and still believes every one of them to be strictly true.

That on the 11th day of October 1872, or two days after receiving the copy of said answer, the complainants' solicitors in said suit requested this deponent's solicitor to keep said answer off the files of the court, until they could offer a certain important suggestion respecting the conduct of the suit; that this suggestion was as follows: That though until lately convinced that deponent's plea regarding his license, namely, the fifth or partial defense hereabout set forth, was not true; they had recently found reason to be less sure on that point, and therefore they desired this deponent to confine his answer in the first instance to said plea only, they agreeing to stipulate that if defeated on that issue, he might then put in and litigate his other defenses.

That deponent with the concurrence of his counsel, refused to take this course; that therefore the complainants' solicitors announced that they intended to admit the truth of said plea of license, and withdraw their suit accordingly, and requested deponent's counsel meantime not to file the proposed answer, as if filed it would operate prejudicially to the business of the complainants by inciting others to litigate the Cummings' patent, and also because the suit would never be decided on the other issues presented, and that thereupon a further indefinite extension of time was given for filing said answer, and negotiations entered into, by virtue of which on the 18th day of October 1872, the said suit was terminated by the two stipulations hereto annexed, marked respectively "Schedule B, stipulations No. 1;" and "Schedule C, stipulation No. 2;" of which the former is on file in said suit, and the latter is in possession of deponent.

Signed, THOS. B. GUNNING.

Subscribed and sworn to before me this 26th day of October, 1872. FRANK RUDD, *Notary Public.*

[NOTORIAL SEAL.] N. Y. County.

---

Schedule B.

U. S. CIRCUIT COURT,
*Southern District of New York.*

The Goodyear Dental Vulcanite Company and Josiah Bacon,

*versus*

Thomas B. Gunning.

It appearing to the complainants that the assignors of the complainants have heretofore entered into an arrangement which equitably entitles the defendant to use the improvement described in the letters patent, for the infringement of which, this suit is brought in his profession as a dentist, on motion of Lee & Alvoid complainants' solicitors, and on the annexed consent of the solicitors of the defendant. It is ordered that the bill in this cause be and the same is hereby dismissed; and that the complainant pay to the defendant his costs to be taxed.

New York, Oct. 18th, 1872. L. B. WOODRUFF,
(A copy.) *Circuit Judge.*

New York, October 18th, 1872.

We consent to the entry of the within order.

RUDD, CASSERLY & MAYOR,
Defendant's Solicitors.

---

Schedule C.

U. S. CIRCUIT COURT,
*Southern District of New York* In equity.

The Goodyear Dental Vulcanite Company and Josiah Bacon, *Complainants,*

*against*

Thomas B. Gunning, *Defendant.*

It is further stipulated herein, in consideration of the settlement of this suit, and of the cessation of litigation thereon:

That the defendant shall not be prosecuted nor held responsible for the use or application of hard rubber or vulcanite, or its equivalents to dental purposes; nor for the use or application of any of the improvements which are the subjects of the letters patent, granted respectively to John A. Cummings, June 7th, 1864; and to John B. Newbrough, as assignee of Robert Hæring, January 19th, 1869, (No. 85,927,) during the term of either of said patents, or during the terms of any reissues or extensions, present or future, of the same, provided such uses or applications be by the said defendant himself, or by such workmen and assistants as he may necessarily employ in his business and practice as a dentist, and be for his own patients only; nor shall he be prosecuted or held responsible for any alleged infringement in the past of either of said letters patent; it being understood that the defendant is not to use, vend or apply under this stipulation more than fifty (50) plates for artificial teeth in any one year,

New York, Oct. 18th, 1872.

Signed, LEE & ALVOID,
Complainants' Solicitors.

Signed, Goodyear Dental Vulcanite Co.
per JOHN H. CHEEVER, President.

