# Erratum

**Published:** 1873-01

**Authors:** 


*Erratum.*—On page 397 of December number, in the article "Cummings' patent, vs. T. B. Gunning," in the 16th line from top of page, the word "red" should be omitted, and the sentence should read, "*the improvement claimed by the patent could not be manufactured so as to be applicable to dental purposes.*

